# Uric acid promotes oxidative stress and enhances vascular endothelial cell apoptosis in rats with middle cerebral artery occlusion

**DOI:** 10.1042/BSR20170939

**Published:** 2018-05-15

**Authors:** Chengfu Song, Xiangdong Zhao

**Affiliations:** Department of Geratology, Yangpu Hospital, Tongji University School of Medicine, Shanghai 200093, China

**Keywords:** middle cerebral artery occlusion, oxidative stress, Uric acid, vascular endothelial cell apoptosis

## Abstract

In patients with cerebral infarction (CI), elevated serum uric acid (UA) level may exacerbate the occurrence and development of carotid atherosclerosis (AS). Our study intended to explore the underlying mechanism. We enrolled 86 patients with CI, and divided them into four groups: Non-AS, AS-mild, AS-moderate, and AS-severe groups; the levels of UA and oxidative stress-related factors in serum were detected. The middle cerebral artery occlusion (MCAO) model was used to stimulate CI in rats, and different doses of UA were administrated. The levels of oxidative stress-related factors in serum were detected. Hematoxylin & eosin (H&E) staining was used to observe the morphological alterations, and the apoptotic cell death detection kit was used to detect apoptotic cells. Increased UA concentration and enhanced oxidative stress were found in AS patients. H&E staining results showed that UA treatment exacerbated morphological damage in rats with MCAO, promoted oxidative stress, and enhanced vascular endothelial cell apoptosis in rats with MCAO.

## Introduction

Cerebral infarction (CI) takes up approximately 60–80% of all strokes and is a common clinical cerebrovascular disease. The mortality rate of CI is just lower than that of cancer and myocardial infarction, ranking third [1]. Atherosclerosis (AS) is the result of a complex interaction between blood elements, disturbed flow, and vessel wall abnormality [2]. Several pathologic processes are involved in atherosclerotic plaque evolution: chronic endothelial injury, with increased permeability, endothelial activation, and monocyte recruitment; cellular growth, with smooth muscle cell migration, proliferation, and extracellular matrix synthesis; degeneration, with lipid accumulation, necrosis, and calcification; inflammation, with leukocyte activation and extracellular matrix degradation; and thrombosis, with platelet recruitment and fibrin formation. In addition, biologic processes may be involved in lesion stabilization or even regression [2]. Increasing studies have indicated the positive relationship between the occurrence of AS and CI [3–7].

Serum uric acid (UA) is the final oxidation product of purine catabolism in humans and primates, and current studies have suggested the relations between serum UA and CI, and its effect on CI [1]. Guo et al. [8] indicated that serum UA is a risk factor for large-artery AS CI. Tao et al. [9] indicated that UA is strongly and closely related to the occurrence and development of carotid AS in patients with CI. Song et al*.* [10] suggested that the rise of serum UA level in patients with CI is closely related to the occurrence of carotid AS and unstable plaque is the most closely related. Thus, it seems that, in patients with CI, elevated UA concentration may exacerbate the occurrence and development of carotid AS. Our study aimed to explore the underlying mechanism.

It is well accepted that oxidative stress plays critical roles in the pathogenesis of AS, since it has the ability to stimulate release of inflammatory cytokines, induce cytotoxicity of blood cells, and induce the production of growth factors [11]. Wang [12] concluded that the relationship between endothelial dysfunction and adverse outcome is likely to be due not only to destabilization of established disease in high-risk populations but also to its impact on the evolution of the atherosclerotic substrate. A previous study also indicated the oxidative stress and endothelial damage in patients with asymptomatic carotid AS [11].Therefore, we speculated that, in patients with CI, elevated UA concentration may exacerbate the occurrence and development of carotid AS by prompting oxidative stress and aggravating endothelial damage.

In our study, we enrolled 86 patients with CI, and divided them into four groups: Non-AS, AS-mild, AS-moderate, and AS-severe groups; the levels of UA and oxidative stress-related factors in serum were detected. The middle cerebral artery occlusion (MCAO) model was used to stimulate CI in rats, and different doses of UA were used.

## Methods

### Patients

The present study enrolled a total of 86 patients with CI (Han nationality), who were received treatment in Yangpu Hospital (Shanghai, China) from December, 2012 to December, 2013. The diagnostic criteria for CI were in accordance with the revised diagnostic criteria of 1995 Fourth National Conference for Cerebrovascular Diseases [13]. All patients were accorded with the diagnostic criteria for cerebrovascular disease and were confirmed by computed tomography (CT) or magnetic resonance imaging (MRI).

The exclusive criteria were: (1) patients with cerebral hemorrhage; (2) patients with cardiogenic cerebral embolism (such as atrial fibrillation and rheumatic heart disease); (3) patients with cerebral arteritis-induced CI; (4) patients with liver and kidney dysfunction, malignant tumors, hyperthyroidism, and acute myocardial infarction. According to the CT examination results, patients were divided into two groups: AS and Non-AS group (*n* = 43 in each group). AS group includes 29 male cases, 14 female cases, with an age range 49–81 years (65 ± 16 years old); Non-AS group includes 31 males and 12 females, with an age range 50–83 years (66.5 ± 16. 5 years old). According to NASCCET classification, vascular atherosclerotic stenosis can be classified as: <29% for mild stenosis (AS-mild group, *n*=15), 30–69% for moderate stenosis (AS-moderate group, *n*=15), and >90% for severe stenosis (AS-severe group, *n*=13) [14]. Thus, in our study, AS were subgrouped into AS-mild, AS-moderate, and AS-severe. Informed consent was obtained from every patient and the present study was approved by the Ethics Committee of Tongji University School of Medicine. All subjects were collected in the morning at 8:30–10:00 for extraction of 2 ml of fasting blood, and kept at room temperature for 30 min. After centrifugation at 3500 rpm for 4 min, serum was collected and stored in −4°C for further analysis.

### Animal experiment

All the experimental protocols were approved by the Animal Ethics Committee of Tongji University School of Medicine. The MCAO model group included 55 SPF-Sprague-Dawley adult male rats (at an age of 8 weeks; with a weight of 250–300 g), which were initially anesthetized by intraperitoneal injection of 10% chloral hydrate (4 ml/kg). The rat was then placed on a wooden shelf and the following procedure was according to Sasaki’s methods [15]. The MCAO model was established by using a suture occluded procedure as follows: the suture was initially inserted into the internal carotid artery, approximately 1.8 cm distal to the bifurcation of internal and external carotid artery. In this place, the suture reached the base of middle cerebral artery (MCA). The suture was then permanently fixed to the internal carotid artery, and the flow of MCA was thus permanently occluded. Eleven additional rats with carotid vascular separation and skin incision were regarded as Sham group in our study.

### UA solution

NaOH (0.4 g) was dissolved in ultrapure water (10 ml) to obtain NaOH solution (1 mM). UA (150 mg) was added subsequently and was dissolved in water, with a final concentration of 15 mg/ml. The solution was filtered using a 0.22-µm filter under a sterile hood, and the solution was stored at room temperature. All were obtained from Sigma (Sigma-Aldrich, St. Louis, MO, U.S.A.).

### Treatment

The rats were housed in a standard animal room under a 12-h light/dark cycle, and were allowed *ad libitum* access to food and water. The temperature and humidity of the animal room were maintained at 25°C and 55% respectively. Rats were received an intragastric administration of UA at 50, 100, 150, and 200 mg/kg body weight twice one day for 12 weeks consecutively (*n* = 11 in each group). Rats treated with normal saline were used as the control group (*n* = 11; MCAO + saline). At 0, 3, 7, and 12 weeks after the experiment, rats were fasted for 14 h and were anesthetized with intraperitoneally injection of chloral hydrate. Intravenous blood (1.5–2 ml) was collected and kept at room temperature for 30 min. After centrifugation at 3500 rpm for 4 min, serum was collected and stored in −4°C for further analysis.

### Measurement of UA, 8-hydroxy-2′-deoxyguanosine (8-OHdG), endothelial nitric-oxide synthase (eNOS), glutathione peroxidase (GSH-Px), and superoxide dismutase (SOD) level in serum

A Hitachi 917 autoanalyzer (Roche Diagnostics, Mannheim, Germany) was used to measure UA in serum samples. Enzyme-linked immunosorbent assay (ELISA) was applied to measure the serum 8-OHdG, following the manufacturer’s instructions (Trevigen, Gaithersburg, MD, U.S.A.). Briefly, sample diluents or standard solution were mixed with a horseradish peroxidase-conjugate reagent. The optical density was read at 450 nm using visible spectrophotometer. The standard curve was generated and the concentration of 8-OHdG in the samples was determined by comparing the OD values of the samples to the standard curve.

ELISA was used to measure the serum eNOS, following the manufacturer’s instructions (Trevigen, Gaithersburg, MD, U.S.A.). Briefly, sample diluents or standard solution were mixed with a horseradish peroxidase-conjugate reagent. The optical density was read at 540 nm using visible spectrophotometer. The standard curve was generated and the concentration of eNOS in the samples was determined by comparing the OD values of the samples to the standard curve.

For serum GSH-Px and SOD assay, GSH-Px and SOD kits were purchased from Nanjing Jiancheng Bioengineering Institute (Jiangsu, China) and following the manufacturer’s instructions. The optical densities were measured at 412 and 550 nm using visible spectrophotometer for GSH-PX and SOD activities respectively. The activities can be expressed as the units per mg of protein sample.

### Measurement of nitric oxide (NO) level in serum

A nitric oxide (NO2^−^/NO3^−^) assay kit (Sigma-Aldrich, U.S.A.) was utilized to measure the total serum NO level. The concentration of NO in serum samples was measured by determining both nitrate and nitrite levels as previously described [16]. Samples were spectrophotometrically quantified using a Turner microplate reader at 540 nm (PROMEGA, U.S.A.).

### Hematoxylin & eosin (H&E) staining

For H&E staining, aorta was separated and was fixed in formalin. The tissues were then embedded in paraffin, and sliced into 5-µm-thick sections. Sections were stained with H&E and visualized under a microscope (Leica DM 2500).

### Cell isolation and culture

Aortic endothelial cells were isolated from rats as previously described [17]. Briefly, rats were anesthetized and killed by rapid cervical dislocation and an incision was quickly made in the abdominal skin, in order to expose the aorta, which was perfused with PBS containing heparin and was then resected. The aortas were placed in Dulbecco’s modified Eagle’s medium (DMEM) and 2% collagenase II solution was injected and maintained inside the aorta for 45 min (Gibco; Thermo Fisher Scientific, Inc., Waltham, MA, U.S.A.). The aortas were then washed with DMEM supplemented with 20% fetal bovine serum (FBS) (Gibco; Thermo Fisher Scientific, Inc.) and endothelial cells were harvested by centrifugation at 2184 rpm for 10 min at 4°C. Subsequently, rat aortic endothelial cells were cultured in DMEM supplemented with 10% FBS, 100 U/ml penicillin, and 100 µg/ml streptomycin in an incubator containing 5% CO_2_ at 37°C.

### Apoptosis assay of vascular endothelial cell

An *in situ* apoptotic cell death detection kit TMR red (Roche Applied Science, Indianapolis, IN) based on a TUNEL assay was used to detect apoptotic cells. The TUNEL assay was performed on cells after 20-h incubation, as per manufacturer’s instruction. By using episcopic-fluorescence microscopy, apoptotic cells were visualized. The apoptotic rate (%) is expressed as the number of apoptotic cells over the total number of cells.

### Statistical analyses

All data analyses were carried out using the GraphPad Prism software (Graph Pad, CA). One-way ANOVA analysis was performed to determine the significant differences. The significance set was adjusted to *P*<0.05.

## Results

### Increased UA concentration and enhanced oxidative stress in AS patients

**Figure 1:**
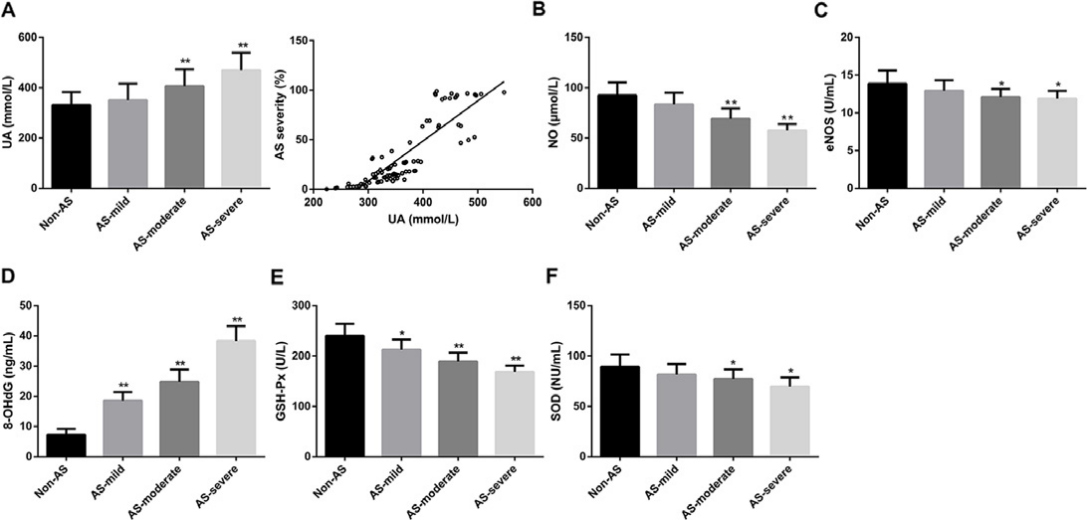
Increased UA concentration and enhanced oxidative stress in AS patients (**A**) The concentrations of UA in Non-AS, AS-mild, AS-moderate, and AS-severe groups and the correlation between UA concentration and AS severity. (**B**) The concentrations of NO in Non-AS, AS-mild, AS-moderate, and AS-severe groups. (**C**) The activities of eNOS in Non-AS, AS-mild, AS-moderate, and AS-severe groups. (**D**) The concentrations of 8-OHdG in Non-AS, AS-mild, AS-moderate, and AS-severe groups. (**E**) The activities of GSH-Px in Non-AS, AS-mild, AS-moderate, and AS-severe groups. (**F**) The activities of SOD in Non-AS, AS-mild, AS-moderate, and AS-severe groups. According to the CT examination results, CI patients were divided into two groups: AS and Non-AS group (*n* = 43 in each group). According to NASCCET classification, vascular atherosclerotic stenosis can be classified as: <29% for mild stenosis (AS-mild, *n*=15), 30–69% for moderate stenosis (AS-moderate, *n*=15), >90% for severe stenosis (AS-severe, *n*=13); *P<0.05 and **P<0.01 compared with Non-AS group.

First, we found that the UA concentration was significantly higher in AS- moderate (*P*<0.01) and AS-severe (*P*<0.01) groups than that in Non-AS group. No significant difference was found between AS-mild and Non-AS groups (Figure 1A). Besides, we found that the UA concentration was positively corrected with the severity of AS (Figure 1A). 8-OHdG is widely used as biomarkers for oxidative stress [18,19]. eNOS generates the endogenous vasodilator NO [20]. GSH-Px and SOD are well accepted as antioxidant enzymes [21]. In our study, the concentration of NO and the activity of eNOS were significantly lower in AS- moderate (*P*<0.01 and *P*<0.01) and AS-severe (*P*<0.05 and *P*<0.05) groups than that in Non-AS groups. No significant difference was found between AS-mild and Non-AS groups (Figure 1B,C). Significant higher concentration of 8-OHdG was found in AS group (AS-mild, *P*<0.01; AS-moderate, *P*<0.01; and AS-severe, *P*<0.01) than that in Non-AS group (Figure 1D). With regard to GSH-Px activity, its activity in AS-mild, AS-moderate, and AS-severe was significantly higher than that in Non-AS group (Figure 1E; *P*<0.05, *P*<0.01, and *P*<0.01 respectively). Besides, SOD activity was significantly lower in AS-moderate (*P*<0.05) and AS-severe (*P*<0.05) groups than that in Non-AS groups. There is no significant difference between AS-mild and Non-AS groups (Figure 1F). Totally, these results showed that increased UA concentration and enhanced oxidative stress were observed in AS patients.

### UA exacerbates morphological damage in rats with MCAO

**Figure 2:**
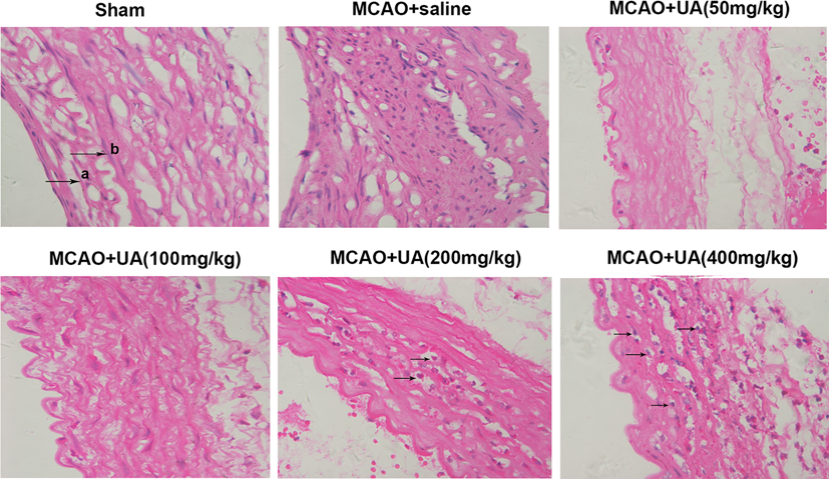
UA exacerbates morphological damage in rats with MCAO UA exacerbated morphological damage in rats with MCAO in a dose-dependent manner. Aorta was separated and was fixed in formalin, then was embedded in paraffin, and sliced into 5-μm-thick sections. Sections were stained with HE and visualized under a microscope (Leica DM 2500). Rats were received an intragastric administration of UA at 50, 100, 150, and 200 mg/kg body weight twice one day for 12 weeks consecutively (*n* = 11 in each group). Rats treated with normal saline were used as the control group (MCAO + saline). a and b in Sham group indicates internal elastic membrane and smooth muscle cell. The arrow in MCAO + UA (200 mg/kg) and MCAO + UA (400 mg/kg) groups indicate foam cells.

As shown in Figure 2, in Sham rats, we found that the vascular wall layer was clear, the intimal surface was smooth and complete, the thickness of smooth muscle layer was normal, and no abnormal changes in the outer layers of connective tissue were observed. In rats with MCAO, the morphological changes were slight as compared with the Sham rats. However, we found that UA exacerbated morphological damage in rats with MCAO in a dose-dependent manner. In MCAO rats that treated with 50 and 100 mg/kg UA, the structure of vascular wall was damaged. In MCAO rats that treated with 200 mg/kg UA, the foam cells were appeared. In MCAO rats that treated with 400 mg/kg UA, the intimal surface appeared significant lesions, a large number of foam cells were appeared in plague, and the structure of vascular wall was damaged. The results were consistent with the morphological characteristics of carotid AS, it may seem that, in rats with MCAO, UA could exacerbate carotid AS progress.

### UA promotes oxidative stress in rats with MCAO

**Figure 3:**
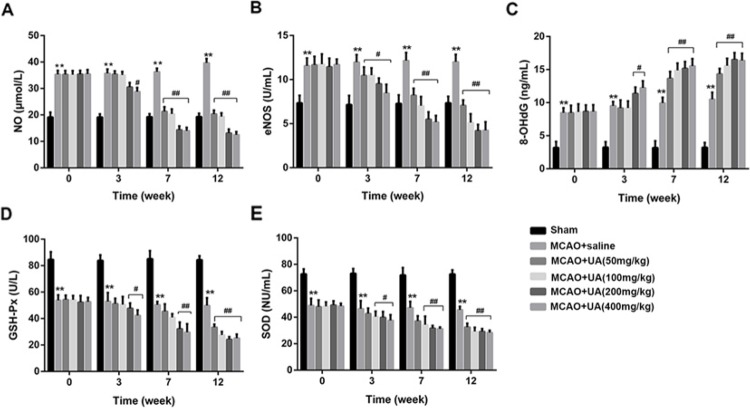
UA promotes oxidative stress in rats with MCAO (**A**) The concentration of UA in each group. (**B**) The activity of eNOS in each group. (**C**) The concentration of 8-OHdG in each group. (**D**) The activity of GSH-Px in each group. (**E**) The activity of SOD in each group. Rats were received an intragastric administration of UA at 50, 100, 150, and 200 mg/kg body weight twice one day for 12 weeks consecutively (*n* = 11 in each group). Rats treated with normal saline were used as the control group (MCAO + saline). **P<0.01 compared with Sham group. ^#^*P*<0.5 and ^##^*P*<0.01 compared with MCAO + saline group.

As shown in Figure 3, the concentration of NO in MCAO was significantly higher than that in the Sham group, and UA treatment caused significant decreased NO concentration both in a time- and dose-dependent manners (Figure 3A). The activity of eNOS in MCAO was significantly higher than that in the Sham group, after UA treatment, significant decreased eNOS activities both in a time- and dose-dependent manners were found (Figure 3B). The concentration of 8-OHdG in MCAO was also significantly higher than that in the Sham group, but UA treatment caused significant increased ROS activity and 8-OHdG concentration both in a time- and dose-dependent manners (Figure 3C). Besides, the activities of GSH-Px and SOD in MCAO were also significantly lower than that in the Sham group, and UA treatment caused significant decreased GSH-Px and SOD activities both in a time- and dose-dependent manners (Figure 3D,E).

### UA enhances vascular endothelial cell apoptosis in rats with MCAO

**Figure 4:**
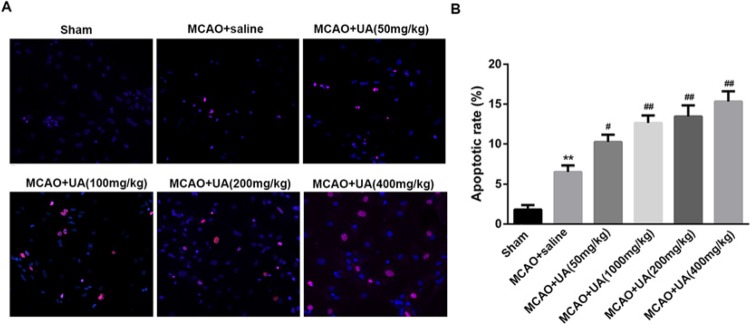
UA enhances vascular endothelial cell apoptosis in rats with MCAO (**A**) The apoptotic cell in each group were visualized under an episcopic-fluorescence microscopy. (**B**) Statistical analysis of the apoptotic cell rate in each group. Aortic endothelial cells were isolated from rats as previously described [17]. **P<0.01 compared with Sham group. ^#^*P*<0.5 and ^##^*P*<0.01 compared with MCAO + saline group. An *in situ* apoptotic cell death detection kit TMR red (Roche Applied Science, Indianapolis, IN) based on a TUNEL assay was used to detect apoptotic cells. The apoptotic rate (%) is expressed as the number of apoptotic cells over the total number of cells. **P<0.01 compared with Sham group. ^#^*P*<0.5 and ^##^*P*<0.01 compared with MCAO + saline group. Red indicates the apoptotic cells and blue indicates nucleus.

To explore whether UA could regulate vascular endothelial cell apoptosis, vascular endothelial cell was isolated from rats with MCAO and the apoptotic assay was performed. As shown in Figure 4, we found the apoptotic cell rate in MCAO group was significantly increased as compared with that in the Sham group. Indeed, UA treatment caused markedly increased apoptotic cell rate in a dose-dependent manner (Figure 4).

## Discussion

In our study, we first found that UA concentration was significantly higher in AS-moderate and AS-severe groups than that in Non-AS group. Chai et al*.* [14] indicated that high serum UA level was correlated with AS stenosis of head and neck vascular in patients with CI. Bei et al*.* [22] indicated that high concentrations of plasma homocysteine and UA are independent risk factors of CI combined with carotid AS, which may play a part in carotid atherosclerosis and plaque formation. Song et al. [10] suggested that the rise of serum UA level in patients with CI is closely related to the occurrence of carotid AS and unstable plaque is the most closely related. To some extent, our study also proved that high serum UA concentration may have severe stenosis.

We found that the concentration of NO, and the activities of eNOS and SOD levels were significantly lower in AS-moderate and AS-severe groups than that in Non-AS groups. Besides, significant higher concentrations of 8-OHdG were found in AS group than that in Non-AS group. And GSH-Px activity in AS group was significantly higher than that in Non-AS group. NO is a potent vasodilator produced by endothelial cells, which exerts an important role in the regulation of regional blood flow and blood pressure, and has the ability to inhibit vascular smooth muscle cell proliferation, as well as inhibit platelet aggregation and leukocyte adhesion to vascular endothelium [23]. Importantly, the complex relationship among nitric oxide (NO)-mediated pathways and atherogenesis has been reported. Due to the numerous pathophysiological actions of NO, abnormalities such as impaired release of NO from the atherosclerotic damaged endothelium and reduction in concentration or activity both of inducible nitric oxide synthase and eNOS could potentially occur [24]. Consistently, in our study, we also found that the level of eNOS was significantly lower in AS-moderate and AS-severe groups than that in Non-AS groups. Ignarro et al. [25] suggested that endothelial damage induced by AS leads to the reduction in bioactivity of eNOS with subsequent impaired release of NO. An important mechanism is local enhanced degradation of NO by increased generation of reactive oxygen species (ROS) and other free radicals, with subsequent cascade of oxidation-sensitive mechanisms in the arterial wall.

It is well known that 8-OHdG is a marker of oxidative stress [26]. Kroese et al*.* concluded that high levels of 8-OHdG in blood and urine are associated with AS and heart failure [27] which is consistent with our results. Differently, 8-OHdG has also been recently rediscovered to inhibit Rac1 in neutrophils and macrophages, thereby inhibiting Rac1-linked functions of these cells, including ROS production through NADPH oxidase activation, phagocytosis, chemotaxis, and cytokine release [26]. In vascular smooth muscle cells, ROS also induce abnormal proliferation and migration leading to progression of AS [26]. Thus, this study showed a novel action of orally active 8-OHdG in suppressing atherosclerotic plaque formation *in vivo* and VSMC activation *in vitro* through inhibition of Rac1. Besides, 8-OHdG was found to prevent plaque formation in a partial ligated apolipoprotein (Apo) E knockout mouse, an acute model of AS [28].

GSH-Px and SOD are two cellular antioxidant enzymatic systems [29]. A decrease in GSH-Px activity was found in cases with severe AS of the coronary artery in comparison with the group with slight or moderate AS [29]. Torzewski et al. [30] revealed that deficiency of GSH-Px accelerates the progression of AS in Apo E-deficient mice, by increasing ROS concentration in the aortic wall as well as increasing overall oxidative stress. Besides, peritoneal macrophages from double-knockout (GPx-1(-/-)ApoE(-/-)) mice showed increased *in vitro* proliferation in response to macrophage-colony-stimulating factor. Also, lower levels of bioactive NO as well as increased tyrosine nitration as a marker of peroxynitrite production were found [30]. Zhang et al*.* [31] suggested that the levels of SOD in AS model group were lower than in normal group. Other study indicated that extracellular SOD is decreased in atherosclerotic patients, and it can be used as a new marker of AS [32]. Our results were consistent with these observations. In MACO rats, we found that UA treatment caused significant low concentration of NO and decreased the activities of eNOS, SOD, and GSH-Px; whereas increased concentration of 8-OHdG. To some extent, these results indicated that UA could exacerbate carotid AS progress by promoting oxidative stress.

In MCAO rats that treated with 400 mg/kg UA, the arterial endothelial cell was shed, the intimal surface was fibrosis and appeared significant lesions, a large number of foam cells were appeared in plague, lipid was deposited, inflammatory cell was aggregated, the tunica media of the vessel wall was calcified, and the three-tier structure of vascular wall was damaged. These results were consistent with the morphological characteristics of carotid AS. It seems that, in rats with MCAO, UA could exacerbate morphological damage. We also found that UA enhances vascular endothelial cell apoptosis in rats with MCAO. Yu et al*.* [33] indicated that UA-induced aging and death of human endothelial cells are medicated by local activation of the renin–angiotensin system and oxidative stress, which provides a novel mechanism of UA-induced endothelial dysfunction, they concluded that therapies targeting UA may be beneficial in cardiovascular disease. A previous study indicated that UA has anti-angiogenic effects (inhibition of endothelial cell migration and proliferation, stimulation of endothelial cell apoptosis) and stimulates proinflammatory mechanisms and pro-oxidative properties that may augment renal injury [34]. Sánchezlozada et al*.* [35] revealed that UA-induced endothelial dysfunction is associated with mitochondrial alterations and decreased intracellular ATP concentrations. Based on results in our study, the deeper and further investigations should be performed to explore the molecular mechanisms. To sum up, our study found increased UA concentration and enhanced oxidative stress in AS patients. UA contributes to the progress of AS in rats with MCAO, not only by promoting oxidative stress, but also enhancing vascular endothelial cell apoptosis in rats with MCAO. Therapies targeting UA may be beneficial in CI patients with AS.

## Abbreviations

AS: atherosclerosis
CI: cerebral infarction
DMEM: Dulbecco’s modified Eagle’s medium
eNOS: endothelial nitric-oxide synthase
GSH-Px: glutathione peroxidase
H&E: hematoxylin & eosin
MCAO: middle cerebral artery occlusion
ROS: reactive oxygen species
SOD: superoxide dismutase
UA: uric acid
